# Genetic Variation among Major Human Geographic Groups Supports a Peculiar Evolutionary Trend in PAX9

**DOI:** 10.1371/journal.pone.0015656

**Published:** 2011-01-27

**Authors:** Vanessa R. Paixão-Côrtes, Diogo Meyer, Tiago V. Pereira, Stéphane Mazières, Jacques Elion, Rajagopal Krishnamoorthy, Marco A. Zago, Wilson A. Silva, Francisco M. Salzano, Maria Cátira Bortolini

**Affiliations:** 1 Departamento de Genética, Instituto de Biociências, Universidade Federal do Rio Grande do Sul, Porto Alegre, Rio Grande do Sul, Brazil; 2 Departamento de Genética e Biologia Evolutiva, Instituto de Biociências, Universidade de São Paulo, São Paulo, São Paulo, Brazil; 3 Departamento de Bioquímica, Universidade Federal de São Paulo, São Paulo, São Paulo, Brazil; 4 Laboratoire d'Anthropobiologie, FRE2960, CNRS, Toulouse, France; 5 Anthropologie Bioculturelle, Faculté de Médecine, CS80011, Marseille, France; 6 Insern, UMR 763, Université Paris Diderot, Hôpital Robert Debré, Paris, France; 7 Departamento de Clínica Médica, Faculdade de Medicina, Universidade de São Paulo, Ribeirão Preto, São Paulo, Brazil; 8 Departamento de Genética, Faculdade de Medicina de Ribeirão Preto, Universidade de São Paulo, Ribeirão Preto, São Paulo, Brazil; University of Utah, United States of America

## Abstract

A total of 172 persons from nine South Amerindian, three African and one Eskimo populations were studied in relation to the *Paired box gene 9* (*PAX9*) exon 3 (138 base pairs) as well as its 5′and 3′flanking intronic segments (232 bp and 220 bp, respectively) and integrated with the information available for the same genetic region from individuals of different geographical origins. Nine mutations were scored in exon 3 and six in its flanking regions; four of them are new South American tribe-specific singletons. Exon3 nucleotide diversity is several orders of magnitude higher than its intronic regions. Additionally, a set of variants in the *PAX9* and 101 other genes related with dentition can define at least some dental morphological differences between Sub-Saharan Africans and non-Africans, probably associated with adaptations after the modern human exodus from Africa. Exon 3 of *PAX9* could be a good molecular example of how evolvability works.

## Introduction

The central goal of genetics is to understand how heritable information encoded in the genome determines the phenotype of an organism [1]. Some results support the idea that morphological evolution can occur basically by a small proportion of major effect mutations in structural, developmental or regulatory key genes (major gene effect hypothesis [2], [3]. Considering this possibility, we studied the entire coding region as well as exon-intron boundaries of the *Paired box homeotic gene 9* (*PAX9*; a transcription gene with a major role in early development, including dentition) to investigate the importance of small changes in major protein-coding genes that could be involved with the normal intra and interspecies dental variation [4].

The *PAX9* coding sequence is divided into four exons. The first exon acts uniquely as the start codon, whereas exon 2 codes for a highly conserved region of the *PAX9* protein, the paired domain, which is responsible for sequence-specific contacts with DNA [5], [ 6], http://www.ensembl.org/index.html. The vital importance of exon 2 is illustrated by an impressive absence of nucleotide sequence variation at this region among 44 species of mammals (V.R. Paixão-Côrtes et al., unpublished data), as well as within and between human populations, and the identification of mutations in the paired domain as etiologic for several cases of both nonsyndromic hypodontia and oligodontia in our species (http://www.ncbi.nlm.nih.gov/omim - OMIM#167416; OMIM#142893). Similarly, although the fourth exon of *PAX9* lacks functional information, it is located in a region which is also relatively conserved across 44 mammalian species (V.R. Paixão-Côrtes et al., unpublished data), and is involved in molar oligodontia in humans, indicating a prominent role of this exon for *PAX9* function [7]. However, the expectation that a similar degree of evolutionary constraint might also be observed at exon 3 of *PAX9* was not confirmed by us [4]. We have obtained evidence not only for a high level of human intraspecific variation, but also that a common polymorphism (*Ala240Pro*; rs4904210) is probably functional and could be associated with third molar agenesis, since *240Pro* homozygotes might present a *PAX9* protein with a slightly reduced DNA-binding capacity [4]. Additionally, our recent [8] as well as other family studies [9], [10], showed that the derived *240Pro* allele has a recessive pattern of inheritance with variable expressivity, since all homozygotes for it had congenital missing third molar(s); the number of missing teeth, however, was different for each affected individual, in agreement with Pereira et al's hypothesis [4].

Here, we presented an analysis based on new *PAX9* exon 3 sequence information and its exon-intron border regions from Native American, African, and Eskimo populations, which were integrated with published information encompassing all major human geographic groups. We also used data from HapMap Phase II [11] to identify signals of possible different genetic backgrounds involved in the dental development in Africans and non-Africans. Finally, we explored the evolutionary scenario related to the origin and prevalence of the *PAX9* polymorphisms, suggesting that exon 3 is an important agent in the ability to evolve (evolvability) of this extremely conserved gene.

## Results

### Mutations and polymorphic sites

A total of 590 base pairs, corresponding to *PAX9* exon 3 and its 5′and 3′ flanking intronic regions were sequenced in 344 human chromosomes, and the data were analyzed together with other previously published sequences [4]. There are four polymorphic sites in the 5′flanking and two in the 3′ flanking intronic regions in the Apalaí, Galibi, Mekranoti, European and African populations (**Table 1**). Only one of these, the variant allele *A* at position 36.205.241 was present in homozygous state in one Galibi individual. As for the sequence changes observed in exon 3, almost all were seen in heterozygosis, two exceptions being variants located at codons 239 and 240 (C→T, *His239His*; and G → C, *Ala240Pro*; see below).

**Table 1:** Mutations observed in PAX9 exon3^a^.

Populations (Numbers below the names indicate the total of chromosomes sequenced) b																							
Mutations					Aché	Apalaí	Arara	Bari	Cayapo	Guarani	Galibi	Jamamadi	Kaigang	Mekranoti	Munducuru	Tenharim	Warao	Xikrin	Yucpa	Eskimo	Japanese	European	African
Region	Position in chrom. 14	SNP	Nucleotide change	Amino acid change	40	18	42	8	2	10	14	10	40	10	16	16	8	34	8	88	28	30	94
5′Flanking	36. 205. 241		C ->A		0	0	0	0	0	0	**2**	0	0	0	0	0	0	0	0	0	0	0	0
	36. 205. 341		G ->A		0	0	0	0	0	0	0	0	0	**1**	0	0	0	0	0	0	0	0	0
	36. 205. 356		G ->C		0	0	0	0	0	0	0	0	0	0	0	0	0	0	0	0	0	**1**	**8**
	36. 205. 362		A ->T		0	**1**	0	0	0	0	0	0	0	0	0	0	0	0	0	0	0	0	0
Exon 3	36. 205. 463		G ->A	Ser226Asn	0	**1**	0	0	0	0	0	0	0	0	0	0	0	0	0	0	0	0	0
	36. 205. 463		G ->T	Ser226Ile	0	0	0	**1**	0	0	0	0	0	0	0	0	0	0	0	0	0	0	0
	36. 205. 492		G ->A	Ala236Thr	0	0	0	0	0	0	0	0	0	0	0	**1**	0	0	0	0	0	0	0
	36. 205. 503	rs12881240	C ->T	His239His	0	0	0	0	0	0	0	0	**1**	0	0	0	0	0	0	**3**	0	**8**	**3**
	36. 205. 504	rs4904210	G ->C	Ala240Pro	0	**2**	**3**	0	0	0	**1**	**4**	**5**	0	**1**	**2**	0	**2**	0	**26**	**4**	**7**	**19**
	36. 205. 504		G ->A	Ala240Thr	0	0	0	0	0	0	0	0	0	0	**1**	**2**	0	**1**	0	0	0	0	0
	36. 205. 510		T ->A	Asn242Asp	0	0	**1**	0	0	0	0	0	0	0	0	0	0	0	0	0	0	0	0
	36. 205. 535		A ->T	Glu250Val	0	**1**	0	0	0	0	0	0	0	0	0	0	0	0	0	0	0	0	0
	36. 205. 537		C ->A	Glu251Lys	0	0	0	0	0	**1**	0	0	0	0	0	0	0	0	0	0	0	0	0
3′Flanking	36. 205.575		G ->A		0	0	0	0	0	0	0	0	0	**0**	0	0	0	0	0	0	0	0	**1**
	36. 205.611		G ->A		0	0	0	0	0	0	0	0	0	**1**	0	0	0	0	0	0	0	0	**1**

a Sequence of 588 bp corresponding to *PAX9* exon 3 (137 bp) and its 5′and 3′ flanking intronic segments (232 bp and 219 bp, respectively). Numbers in columns 6–24 are those observed for the derived allele in each population.

b Sources: Present study, Apalaí, Arara, Cayapo, Galibí, Jamamadí, Mekranoti, Munducuru, Tenharim, Xikrin, Eskimo, and Africans. Aché, Bari, Guarani, Kaingang, Warao, Yucpa, Japanese, and Europeans [4].

We found several singletons in exon 3, representing new South American tribe-specific variants. They were the G → A (*Ser226Asn*) transition (Apalaí), which is located in the same position as a previously described polymorphism found in the Bari from Venezuela (G →T, *Ser226Ile*); the G →A (*Ala236Thr*) transition (Tenharim); the T → A (*Asn242Asp*) transversion (Arara); and the A → T (*Glu250Val*) transversion (Apalaí; **Table 1**). Another C → A (*Glu251Lys*) mutation, previously seen exclusively in the Guarani [4] was not observed in the present investigation. On the other hand, the derived *T* allele of the *His239His* single nucleotide polymorphism (SNP) previously observed only in the Kaingang of southern Brazil [4] was now also found among the Eskimo. Since this variant was also observed with relatively high frequencies in European (27%) and African (3%) populations, its presence among the Kaingang and Eskimo could be due to admixture with non-Amerindians.

The substitution of a G for A at chromosome 14 position 36.205.504, which results in an amino acid change at residue 240, was detected in the Mundurucu, Tenharim and Xikrin. Curiously, in this same position, variant C of the *Ala240Pro* SNP can be found. Our results indicate that the *C/Pro* allele is widely distributed in South Amerindians living in distant scattered geographic regions and speaking different linguistic stocks, as well as in Eskimos, suggesting that it came from Asia to the New World with the first migrants. *Ala240Thr*, on the other hand, seems to be an American autochthonous mutation, but with relatively ancient origin, since it is present in individuals who speak languages classified in the two major South Amerindian linguistic families (Tupian and Jêan; split at least ∼5 thousand years before present [12]. Finally, allele *T* at the *His239His* SNP is only present in populations where the *Ala240Pro* allele *C* is also found (**Table 1**), but they occur in different individuals, indicating that the derived alleles, *239-T* and *240-C*, are not in linkage disequilibrium.

To better explore the results obtained with the *Ala240Pro* SNP, detailed information on its genotype and allele frequencies is given in **Table 2**. Although some sample sizes are small, *C/Pro* frequencies vary considerably over the 15 South American tribes analyzed: values range from zero (in seven populations) to 40% (Jamamadi). The highest and lowest *C/Pro* frequencies can be found in Asia (45%) and Africa (20%), respectively, while in Eskimos and Europeans (30%) the values are intermediate [4], [ 13, present study].

**Table 2:** Genotype and allele distributions of the Ala240Pro polymorphism in 15 South Amerindian and other human populations^a^.

Population	Number of individuals	Genotype frequency			Allele frequency		References
		*Ala/Ala G/G(%)*	*Ala/Pro G/C (%)*	*Pro/Pro C/C (%)*	*G (Ala)*	*C (Pro)*	
*South Amerindian*							
Aché	20	20 (100)	0	0	1	0	[4]
Apalaí	9	7 (78)	2 (22)	0	0.89	0.11	Present study
Arara	21	18 (86)	3 (14)	0	0.86	0.14	Present study
Bari	4	4 (100)	0	0	1	0	[4]
Cayapó	1	1 (100)	0	0	1	0	Present study
Galibi	7	6 (86)	1 (14)	0	0.86	0.14	Present study
Guarani	5	5 (100)	0	0	1	0	[4]
Jamamadi	5	2 (40)	2(40)	1(20)	0.6	0.40	Present study
Kaigang	20	15 (75)	5 (25)	0	0.88	0.12	[4]
Mekranoti	5	5(100)	0	0	1	0	Present study
Munducuru	8	7 (88)	1(12)	0	0.94	0.06	Present study
Tenharim	8	6 (75)	2 (25)	0	0.88	0.12	Present study
Warao	4	4 (100)	0	0	1	0	[4]
Xikrin	17	15 (88)	2 (12)	0	0.94	0.06	Present study
Yucpa	4	4 (100)	0	0	1	0	[4]
Total	138	119 (86)	18 (13)	1 (<1)	0.91	0.08	
							
Eskimo	44	21(48)	20(45)	3 (7)	0.70	0.30	Present study
Asians^b^	116	40 (35)	47 (40)	29 (25)	0.55	0.45	[4], [15]
Europeans^c^	365*	169 (46)	172 (47)	24 (7)	0.70	0.30	[4]
Africans	47	29 (62)	17 (36)	1 (2)	0.80	0.20	Present study

a Data obtained from RFLP or sequencing;

b Chinese and Japanese;

c Spaniards and Polish.

### Genetic variability

Nucleotide diversity (π) values for *PAX9* exon 3 and its intronic flanking regions are summarized in **Table S1**. Considering exon 3 only, values for π (x 10^4^) for the Amerindian sample as a whole (16.4) are lower than those obtained for the Eskimos (37.2), Europeans (56.1), and Africans (28.1). Curiously the African value is lower than that observed for Europeans, contrasting with studies using neutral markers [14]. Table S1 also shows that the π values for exon 3 are generally several orders of magnitude higher than those calculated for the non-coding regions in all populations, indicating a reduced evolutionary constraint.

### Demographic simulations and neutral/natural selection tests using *PAX9* human data

To better investigate the possibility of departures from a standard neutral model with the *PAX9* human data, we used Tajima's D, and Fu and Li's D* and F* statistics, and afterwards considered them under the assumption of several demographic scenarios, since population history and demography can result in both negative or positive values for these statistics. The strong negative values found for South Amerindians (**Table 3**) are probably determined by demographic processes, since their significance is lost when scenarios of bottlenecks with later expansion are simulated (**Table S2**). Note, however, that for the remaining groups several values are positive and statistically significant under the different population growth scenarios, considering the exon 3 datasets only (**Tables 3** **and S2**). Positive values for these statistics generally indicate an excess of intermediate frequency alleles which is incompatible with a simple neutral model.

**Table 3:** Statistical tests to detect departures from a standard neutral model a.

Population	Tajima's D (Non -Coding)	Tajima's D (Exon 3)	Fu and Li's test (Non -Coding)		Fu and Li's test (Exon 3)	
			D*	F*	D*	F*
South Amerindians	−2,13561*	−1,84693 *	−3,84723*	−2,13561*	−3,03182**	−3,08405**z
Eskimo	0	0,46730	0	ND	0,69567	0,73072
Japanese	0	−0,01865	0	ND	0,60275	0,49703
Europeans	−1,14700	1,09500	−1,68214	−1,76554	0,80615	1,02510
Africans	−1,19605	−0,01004	−1,97525	−2,02829	0,69032	0,55518

a Sequence data from the present study and [4]. Statistical significance:

*P<0.05;

**P<0.02, ND: Not determined.

### Third molar agenesis and the Ala240Pro polymorphism

The number of *CC/ProPro* homozygotes (2%) observed in our Sub-Saharan samples is compatible with the frequency of agenesis in people that live in this African region (∼2%, excluding Khoi-San and Pygmy[15]–[17]). On the other hand, the frequency of *CC/ProPro* homozygotes alone (7%, 7%, 25%) cannot explain the level of third molar agenesis among Eskimos (∼18% [18]), Europeans (∼20%; [17], [19], [20]) and Asians (47% [21]). In fact, our familial study [8] indicated that *GG/ArgArg* homozygotes also display third molar agenesis. Anthropological studies conducted with Native Americans, on the other hand, reveal that it is difficult to establish a general third molar agenesis pattern for Native Americans as a whole, due to the large amount of variation found (from zero to ∼ 40% [18], [22]–[25]. It seems that the *Ala240Pro* polymorphism has little influence in the level of third molar agenesis detected in Amerindian populations.

### The search for distinct gene dental complexes using the HapMap panel

As indicated above, other genetic variants besides those of *PAX9* should influence the dental, jaw and maxillary development in different human populations. We have therefore searched for possible distinct continental gene dental complexes using information from Africans, Europeans and Asians. Unfortunately, the available data for Native Americans are not sufficient to include them in the analysis. Data from 102 dental development genes and their adjacent regions were considered (release 21 - http://hapmap.ncbi.nlm.nih.gov/ [11]). **Table S3** lists these genes and **Table S4** the traits that are associated to them. The frequencies of 6,001 SNPs distributed along a 10 Mbp of the indicated region was then obtained and *F_ST_* values for African/European, African/East Asian, and European/East Asian comparisons were calculated. Higher *F_ST_* values (1.3%≥0.6) were observed when Africans were compared with Europeans or Asians. The former number is significantly higher than that estimated by Wu and Zhang [26] using a random gene panel. This excess of high *F_ST_* values can be a signal for natural selection, since population-specific positive selection tends to increase *F_ST_* [27].

Additionally, a high proportion (65–70%) of extreme integrated haplotype score (iHS) negative values (<−3.0), for the top score *PAX9* SNPs was observed for Asians and Europeans (**Table S5**), while in Africans, SNPs with extreme iHS negative values represented only 30% of the cases. An excess of extreme iHS negative values can indicate that the derived alleles (or haplotypes) have swept up in frequency, an indication of positive selection [28]. On the other hand, the extreme iHS positive values for Africans occur in 70% of the cases. This indicates that the ancestral alleles have been fixed preferentially in Africa [28].

These results reinforce the idea that *PAX9* is not the unique gene involved in the dental development and that only an approach based on gene network can lead to a more comprehensive view of the complex biology involved.

## Discussion

Even considering that many polymorphisms are eliminated or fixed as a consequence of genetic drift, it is expected that part of the human molecular diversity is a result of variable selective pressures in different geographical regions, each with its own different climate, pathogens and sources of food [29]–[32]. Additionally, natural selection may act differently in distinct genome segments. While portions of a given gene, for example, are largely invariable due to a strong purifying selection, other regions may vary due to positive selection, relaxation of purifying selection, and/or balancing selection [33], [34]. The present work illustrates this concept, demonstrating that the human *PAX9* might be a good example of these interpopulation and intragene evolutionary dynamics.

By analyzing genetic variation in several Native American tribes, one Eskimo population and other major human groups, we provide evidence for higher nucleotide diversity in exon 3 than in the non-coding sequences of the human *PAX9*. Given the features previously associated with other segments of *PAX9* (i.e. a high degree of conservation due to strong purifying selection in exons as well as in intronic regions across species [4], a pertinent question is why the third exon of this gene possesses such dissimilar pattern of nucleotide diversity when compared to other coding and non-coding regions.

In about 60,000 years, modern humans left Africa and colonized all other continents, with the exception of Antarctica [35], [36]. Since then, human populations have lived in an extraordinary variety of habitats. Additionally, for many human populations, the diet and life style changed drastically due to cultural practices. In this context, a lower number of teeth could be advantageous in populations with progressive reduction of the dental arches and a diet based on processed food, since it would prevent impacted and defective third molars, as well as related-oral problems, an important cause of morbidity in non-industrial societies [4], [37]. Since *PAX9* has a crucial role in skeleton, thymus and parathyroid glands organogenesis [38]–[40] it is valid to speculate that the high level of diversity in only one specific region of the gene might offer options for selection in a context as those resulting from dispersal to new habitats or rapid cultural changes. Biologists have increasingly been asking whether evolvability, the ability of a biological system to evolve, itself evolves, and whether this phenomenon is a result of natural selection or a by- product of other evolutionary processes [41]. *PAX9* exon 3 could be a good molecular example as how evolvability develops, since it may have been an alternative to promote functional adaptations in human dentition. Our results, based on demographic simulations and neutral selection tests, showed that the variation found in *PAX9* exon 3 is incompatible with a simple neutral model, being consistent with the fact that at least one variant at this exon (*Ala240Pro*) might be functional.

Finally, our databank analysis detected a complex involving *PAX9* and 101 other genes related to dentition that can be under different patterns of evolution in Africa and in other continents, defining at least some dental morphological differences between Sub-Saharan Africans and non-Africans, probably associated with adaptations after the modern human exodus from Africa.

## Methods

### Populations examined

*PAX9* exon 3 and its flanking intronic sequences were obtained from 172 individuals from nine South Amerindian (N = 81) one Siberian Eskimo (N = 44) and a composite Sub-Saharan African sample (N = 47). Nucleotide sequences of the same DNA region from 15 Europeans, 14 Japanese and 57 South Amerindians were re-analyzed based on our previous investigations [4]. Additional data for the *Ala240Pro* (rs4904210) polymorphism were obtained through HAPMAP (http://www.hapmap.org/cgi-perl/ [11]) and dbSNP (http://www.ncbi.nlm.nih.gov/projects/SNP/) databases. Relevant information about the new populations studied is provided in Supplemental Material **Table S6**.

Ethical approval for the present study was provided by the Brazilian National Ethics Commission (CONEP Resolution no. 123/98) for the Amerindian and Siberian samples, as well as by ethics committee of Hôpital Robert Debré, Paris France for the African ones. Informed oral consent was obtained from all participants, since they were illiterate, and they were obtained according to the Helsinki Declaration. The ethics committees approved the oral consent procedure.

### DNA extraction, PCR amplification and sequencing

Genomic DNA was extracted from plasma and glycerolized red blood cells stored in our laboratory as a result of previous studies, listed in **Table S6**, using the QIAamp DNA MiniKit (Qiagen) and following the manufacturer's instructions. Non-Brazilian samples were collected and processed as indicated in the references given in **Table S6**.

*PAX9* exon 3 (137 bp) and its 5′and 3′ flanking intronic segments (232 bp and 219 bp, respectively) were amplified using primers and conditions described elsewhere [4]. The PCR products were purified following the Exonuclease I and Alkaline Phosphatase (Amersham Biosciences - GE Healthcare) protocols. Both DNA strands were sequenced using MegaBace500 and MegaBace1000 (Amersham Biosciences - GE Healthcare) sequencing machines. Whenever a putative mutation was identified, the sample was re-amplified and re-sequenced for confirmation.

### Data analysis

Sequences were aligned and their quality as well as the assessment of the accuracy of the resulting data, were obtained using the Phred, Phrap and Consed programs (http://www.genome.washington.edu). Additionally, all chromatograms were visualized and checked manually.

For all groups with exon 3 *PAX9* sequences available one population parameter was estimated using Arlequin 3.11 (http://cmpg.unibe.ch/software/arlequin3/): nucleotide diversity (π) [2] To detect departures from a standard neutral model, we used Tajima's D [42] and Fu and Li's D* and F* [43] tests (implemented using Mega 4.0 [44] and DnaSP 4.10.9 [45]. Significantly positive and negative values for these statistics lead to the rejection of the null hypothesis of mutation-drift equilibrium. However, since it is well-known that these parameters are also affected by the demographic histories of the population considered [29], we incorporated in the analysis coalescence simulations using Rogers' algorithm [46] as implemented by [47] in the DFSC 1.1 software (www.xmission.com/~wooding/DFSC/). Distinct scenarios on times of expansion and initial population sizes compatible with the available data were simulated as follows: 1) For all samples, initial populations of 1,000 or 10,000 individuals [48], [49] starting 60,000 years ago [35], [36], [49]; 2) For all non-African populations, initial population of 10,000, date 40,000 years ago; 3) For Japanese only, 3,000 starting individuals, date 37,000 years ago [50]; 4) For the Native American and Eskimo dataset, initial populations of 80 or 500 individuals [51], [52], starting 18,000 years ago [51], [53]. For all scenarios described above we considered assumptions of population growth ranging from 1-fold (stationary) to 100-fold. The substitution rate (µ) used to obtain the θ values under the different scenarios was 1×10^−9^ [47].

Based on the approach used by Wu and Zhang [26], we also searched a set of genes related with dental development using the Gene Ontology database (browser AmiGO - http://amigo.geneontology.org/cgi-bin/amigo/browse.cgi [54]). This procedure allowed the selection of 102 genes (including *PAX9*) located in the autosomes (**Table S3**). The function of these genes was verified using GeneDeks V3 software (http://www.genecards.org/ [55]- **Table S4**). The major trait phenotype associated to these genes was growth/size (63,11%) followed by craniofacial characteristics (57,28%). A region covering 10 Mbp, upstream of the 5′ end and downstream of the 3′ terminus for each of the 102 genes was used to select SNPs, and a total of 6,001 SNPs were selected. Their allele frequencies were retrieved from HapMap Phase II (release 21 - http://hapmap.ncbi.nlm.nih.gov/ [11]) from three populations: African (YRI panel including 60 Yoruban individuals from Ibadan), European (CEU panel including 60 Utah residents with northern and western Europe ancestry) and 89 East Asian (ASN panels including Han Chinese- HCB and Japanese from Tokyo-JPT). Unfortunately, the rs4904210 *Ala240Pro* polymorphism is not included in these panels. F_ST_ values between African (YRI)/European (CEU), African (YRI)/East Asian (EA) and European (CEU)/East Asian (EA), as well as iHS (integrated haplotype score statistic, used for scanning single nucleotide polymorphism data for signals of selection [28] were recovered from the Haplotter http://haplotter.uchicago.edu/selection/ and SNP@Evolution (http://bighapmap.big.ac.cn/ [56]) databases.

## Supporting Information

Table S1 Additional information’s about mutations and nucleotide sequence diversity estimates of *PAX9* exon 3 and non-exon 3 bordering regions in several human populations.
(DOC)

Table S2 **P values of Tajima's D, and Fu and Lís D and F statistics calculated under several demographic scenarios**
(XLS)

Table S3 **List of dental development genes.**
(XLS)

Table S4 **Traits associated to 102 genes**
(XLS)

Table S5 Integrated haplotype score (iHS) values for the top score SNPs of *PAX9*
(DOC)

Table S6 Sample sizes, geographic location, and linguistic information on fifteen South Amerindian, one Eskimo and one composite African populations investigated in this study
(DOC)
