# Supplementary material for: Genetic Variation among Major Human Geographic Groups Supports a Peculiar Evolutionary Trend in PAX9
Source: PLoS One. 2011 Jan 27;6(1):e15656. doi: 10.1371/journal.pone.0015656 (PMC3029280; doi:10.1371/journal.pone.0015656)
Supplement: Table S1 — Additional information’s about mutations and nucleotide sequence diversity estimates of PAX9 exon 3 and non-exon 3 bordering regions in several human populations. (DOC) [file pone.0015656.s001.doc]

| **Table S1.** Additional information’s about mutations and nucleotide sequence diversity estimates of *PAX9* exon 3 and non-exon 3 bordering regions in several human populations. | | | | | | |
| --- | --- | --- | --- | --- | --- | --- |
| **Population** | **Region** | **Number of sequences** | **Number of sites** | **Number of mutations** | **Number of non-synonymous mutations** | **π± SE** |
| Aché |  | 40 |  |  |  |  |
|  | Exon 3 |  | 138 | 0 | 0 | 0 |
|  | Non-coding |  | 452 | 0 |  | 0 |
|  | Total |  | 590 | 0 |  | 0 |
| Apalaí |  | 18 |  |  |  |  |
|  | Exon 3 |  | 138 | 3 | 3 | 0.00313 ± 0.00330 |
|  | Non-coding |  | 452 | 1 |  | 0.00025 ± 0.00047 |
|  | Total |  | 590 | 4 |  | 0.00092 ± 0.00089 |
| Arara |  | 42 |  |  |  |  |
|  | Exon 3 |  | 138 | 2 | 2 | 0.00162 ± 0.00217 |
|  | Non-coding |  | 452 | 0 |  | 0 |
|  | Total |  | 590 | 2 |  | 0.00038 ± 0.00051 |
| Bari |  | 8 |  |  |  |  |
|  | Exon 3 |  | 138 | 1 | 1 | 0.00311 ± 0.00354 |
|  | Non-coding |  | 452 | 0 |  | 0 |
|  | Total |  | 590 | 1 |  | 0.00073 ± 0.00083 |
| Cayapo |  | 2 |  |  |  |  |
|  | Exon 3 |  | 138 | 0 | 0 | 0 |
|  | Non-coding |  | 452 | 0 |  | 0 |
|  | Total |  | 590 | 0 |  | 0 |
| Galibi |  | 14 |  |  |  |  |
|  | Exon 3 |  | 138 | 1 | 1 | 0.00191 ± 0.00250 |
|  | Non-coding |  | 452 | 1 |  | 0.00058 ± 0.00076 |
|  | Total |  | 590 | 2 |  | 0.00089 ± 0.00089 |
| Guarani |  | 10 |  |  |  |  |
|  | Exon 3 |  | 138 | 1 | 1 | 0.00258 ± 0.00307 |
|  | Non-coding |  | 452 | 0 |  | 0 |
|  | Total |  | 590 | 1 |  | 0.00060 ± 0.00072 |
| Jamamadi |  | 10 |  |  |  |  |
|  | Exon 3 |  | 138 | 1 | 1 | 0.00386 ± 0.00398 |
|  | Non-coding |  | 452 | 0 |  | 0 |
|  | Total |  | 590 | 1 |  | 0.00090 ± 0.00092 |
| Kaingang |  | 40 |  |  |  |  |
|  | Exon 3 |  | 138 | 2 | 1 | 0.00199 ± 0.00244 |
|  | Non-coding |  | 452 | 0 |  | 0 |
|  | Total |  | 590 | 2 |  | 0.00046 ± 0.00057 |
| Mekranoti |  | 10 |  |  |  |  |
|  | Exon 3 |  | 138 | 0 | 0 | 0 |
|  | Non-coding |  | 452 | 2 |  | 0.00088 ± 0.00101 |
|  | Total |  | 590 | 2 |  | 0.00068 ± 0.00077 |
| Munducuru |  | 16 |  |  |  |  |
|  | Exon 3 |  | 138 | 2 | 2 | 0.00175 ± 0.00236 |
|  | Non-coding |  | 452 | 0 |  | 0 |
|  | Total |  | 590 | 2 |  | 0.00041 ± 0.00055 |
| Tenharim |  | 16 |  |  |  |  |
|  | Exon 3 |  | 138 | 2 | 2 | 0.00326 ± 0.00341 |
|  | Non-coding |  | 452 | 0 |  | 0 |
|  | Total |  | 590 | 2 |  | 0.00076 ± 0.00080 |
| Warao |  | 8 |  |  |  |  |
|  | Exon 3 |  | 138 | 0 |  | 0 |
|  | Non-coding |  | 452 | 0 |  | 0 |
|  | Total |  | 590 | 0 |  | 0 |
| Xikrin |  | 34 |  |  |  |  |
|  | Exon 3 |  | 138 | 2 | 2 | 0.00084 ± 0.00152 |
|  | Non-coding |  | 452 | 0 |  | 0 |
|  | Total |  | 590 | 2 |  | 0.00020 ± 0.00036 |
| Yupca |  | 8 |  |  |  |  |
|  | Exon 3 |  | 138 | 0 |  | 0 |
|  | Non-coding |  | 452 | 0 |  | 0 |
|  | Total |  | 590 | 0 |  | 0 |
| **Table S1.** Cont. |  |  |  |  |  |  |
| **Population** | **Region** | **Number of sequences** | **Number of sites** | **Number of mutations** | **Number of non-synonymous mutations** | **π ± SE** |
| South Amerindians  (Average) |  | 276 |  |  |  |  |
|  | Exon 3 |  | 138 | 9 | 8 | 0.00164 ± 0.00215 |
|  | Non-coding |  | 452 | 4 |  | 0.00008 ± 0.00025 |
|  | Total |  | 590 | 13 |  | 0.00045 ± 0.00055 |
| Eskimo |  | 88 |  |  |  |  |
|  | Exon 3 |  | 138 | 2 | 1 | 0.00372 ± 0.00351 |
|  | Non-coding |  | 220 | 0 |  | 0 |
|  | Total |  | 590 | 2 |  | 0.00087 ± 0.00082 |
| Asians  (Japanese) |  | 28 |  |  |  |  |
|  | Exon 3 |  | 138 | 1 | 1 | 0.00184 ± 0.00236 |
|  | Non-coding |  | 452 | 0 |  | 0 |
|  | Total |  | 590 | 1 |  | 0.00043 ± 0.00055 |
| Europeans  (Polish/Spaniard) |  | 30 |  |  |  |  |
|  | Exon 3 |  | 138 | 1 | 1 | 0.00561 ± 0.00469 |
|  | Non-coding |  | 452 | 1 |  | 0.00014 ± 0.00034 |
|  | Total |  | 590 | 2 |  | 0.00143 ± 0.00116 |
| Africans |  | 94 |  |  |  |  |
|  | Exon 3 |  | 138 | 2 | 1 | 0.00281± 0.000042 |
|  | Non-coding |  | 452 | 2 |  | 0.00044 ± 0.000012 |
|  | Total |  | 590 | 4 |  | 0.00100± 0.00013 |
| All |  | 516 |  |  |  |  |
|  | Exon 3 |  | 138 | 17 | 13 | 0.00262± 0.000020 |
|  | Non-coding |  | 452 | 7 |  | 0.00014± 0.00003 |
|  | Total |  | 590 | 24 |  | 0.00072± 0.00005 |
| . | | | | | | |
