# Supplementary material for: Genetic Variation among Major Human Geographic Groups Supports a Peculiar Evolutionary Trend in PAX9
Source: PLoS One. 2011 Jan 27;6(1):e15656. doi: 10.1371/journal.pone.0015656 (PMC3029280; doi:10.1371/journal.pone.0015656)
Supplement: Table S5 — Integrated haplotype score (iHS) values for the top score SNPs of PAX9 (DOC) [file pone.0015656.s005.doc]

| **Table S5.** Integrated haplotype score (iHS)^a^ values for the top score SNPs of *PAX9*. | | | | | | | | | | | | | | | | | | |
| --- | --- | --- | --- | --- | --- | --- | --- | --- | --- | --- | --- | --- | --- | --- | --- | --- | --- | --- |
| **GENE** | **European (CEU)** | | | | | | **African (YRU)** | | | | | | **Asian (ASN)** | | | | | |
|  | SNP | Physical location | Derived allele frequency | **iHS** | iHS/ASN | iHS/YRU | SNP | Physical location | Derived allele frequency | **iHS** | iHS/ASN | iHS/CEU | SNP | Physical location | Derived allele frequency | **iHS** | iHS/YRU | iHS/CEU |
| ***PAX9*** | [rs723204](http://hg-wen.uchicago.edu/cgi-bin/ihh.cgi?target=rs723204&chr=14&db=ceu&sz=4&action=1&ht=0&ver=2) | 35441383 | 0.083 | **3.531** | 1.0880 | 2.0703 | [rs7146383](http://hg-wen.uchicago.edu/cgi-bin/ihh.cgi?target=rs7146383&chr=14&db=yri&sz=4&action=1&ht=0&ver=2) | 31382048 | 0.300 | **4.252** | 1.000 | 1.000 | [rs1349124](http://hg-wen.uchicago.edu/cgi-bin/ihh.cgi?target=rs1349124&chr=14&db=asn&sz=4&action=1&ht=0&ver=2) | 40409503 | 0.094 | **-3.529** | ND | -0.4104 |
|  | [rs766056](http://hg-wen.uchicago.edu/cgi-bin/ihh.cgi?target=rs766056&chr=14&db=ceu&sz=4&action=1&ht=0&ver=2) | 35437846 | 0.083 | **3.505** | 1.0642 | 2.064 | [rs8005763](http://hg-wen.uchicago.edu/cgi-bin/ihh.cgi?target=rs8005763&chr=14&db=yri&sz=4&action=1&ht=0&ver=2) | 31296963 | 0.692 | **-4.154** | ND | ND | [rs7156770](http://hg-wen.uchicago.edu/cgi-bin/ihh.cgi?target=rs7156770&chr=14&db=asn&sz=4&action=1&ht=0&ver=2) | 34614051 | 0.950 | **3.383** | 1.0050 | 0.4925 |
|  | [rs1169007](http://hg-wen.uchicago.edu/cgi-bin/ihh.cgi?target=rs1169007&chr=14&db=ceu&sz=4&action=1&ht=0&ver=2) | 35431912 | 0.092 | **3.355** | 1.0893 | 1.6048 | [rs2022738](http://hg-wen.uchicago.edu/cgi-bin/ihh.cgi?target=rs2022738&chr=14&db=yri&sz=4&action=1&ht=0&ver=2) | 35163591 | 0.167 | **-4.147** | ND | 0.4155 | [rs8019741](http://hg-wen.uchicago.edu/cgi-bin/ihh.cgi?target=rs8019741&chr=14&db=asn&sz=4&action=1&ht=0&ver=2) | 34593017 | 0.950 | **3.359** | ND | 0.6437 |
|  | [rs12883235](http://hg-wen.uchicago.edu/cgi-bin/ihh.cgi?target=rs12883235&chr=14&db=ceu&sz=4&action=1&ht=0&ver=2) | 32753206 | 0.133 | **-3.260** | ND | ND | [rs7143532](http://hg-wen.uchicago.edu/cgi-bin/ihh.cgi?target=rs7143532&chr=14&db=yri&sz=4&action=1&ht=0&ver=2) | 31355122 | 0.275 | **4.093** | ND | ND | [rs8011060](http://hg-wen.uchicago.edu/cgi-bin/ihh.cgi?target=rs8011060&chr=14&db=asn&sz=4&action=1&ht=0&ver=2) | 34616124 | 0.950 | **3.305** | 0.9911 | 0.5050 |
|  | [rs17556373](http://hg-wen.uchicago.edu/cgi-bin/ihh.cgi?target=rs17556373&chr=14&db=ceu&sz=4&action=1&ht=0&ver=2) | 32753590 | 0.133 | **-3.254** | ND | ND | [rs11625294](http://hg-wen.uchicago.edu/cgi-bin/ihh.cgi?target=rs11625294&chr=14&db=yri&sz=4&action=1&ht=0&ver=2) | 35143319 | 0.183 | **-3.999** | ND | 0.1066 | [rs17111276](http://hg-wen.uchicago.edu/cgi-bin/ihh.cgi?target=rs17111276&chr=14&db=asn&sz=4&action=1&ht=0&ver=2) | 40518850 | 0.161 | **-3.221** | ND | ND |
|  | [rs10483431](http://hg-wen.uchicago.edu/cgi-bin/ihh.cgi?target=rs10483431&chr=14&db=ceu&sz=4&action=1&ht=0&ver=2) | 32751215 | 0.133 | **-3.228** | ND | ND | [rs3985198](http://hg-wen.uchicago.edu/cgi-bin/ihh.cgi?target=rs3985198&chr=14&db=yri&sz=4&action=1&ht=0&ver=2) | 40557969 | 0.625 | **3.921** | 2.6532 | 0.6225 | [rs10130324](http://hg-wen.uchicago.edu/cgi-bin/ihh.cgi?target=rs10130324&chr=14&db=asn&sz=4&action=1&ht=0&ver=2) | 39188660 | 0.400 | **-3.189** | ND | ND |
|  | [rs7155975](http://hg-wen.uchicago.edu/cgi-bin/ihh.cgi?target=rs7155975&chr=14&db=ceu&sz=4&action=1&ht=0&ver=2) | 32751670 | 0.133 | **-3.227** | ND | ND | [rs8020397](http://hg-wen.uchicago.edu/cgi-bin/ihh.cgi?target=rs8020397&chr=14&db=yri&sz=4&action=1&ht=0&ver=2) | 31265993 | 0.267 | **3.809** | 0.4679 | 1.0533 | [rs17111338](http://hg-wen.uchicago.edu/cgi-bin/ihh.cgi?target=rs17111338&chr=14&db=asn&sz=4&action=1&ht=0&ver=2) | 40564605 | 0.161 | **-3.134** | ND | -0.6263 |
|  | [rs17471435](http://hg-wen.uchicago.edu/cgi-bin/ihh.cgi?target=rs17471435&chr=14&db=ceu&sz=4&action=1&ht=0&ver=2) | 32752876 | 0.133 | **-3.219** | ND | ND | [rs10141958](http://hg-wen.uchicago.edu/cgi-bin/ihh.cgi?target=rs10141958&chr=14&db=yri&sz=4&action=1&ht=0&ver=2) | 31992333 | 0.150 | **3.800** | ND | ND | [rs7160889](http://hg-wen.uchicago.edu/cgi-bin/ihh.cgi?target=rs7160889&chr=14&db=asn&sz=4&action=1&ht=0&ver=2) | 34605736 | 0.050 | **-3.076** | -0.3874 | ND |
|  | [rs7161508](http://hg-wen.uchicago.edu/cgi-bin/ihh.cgi?target=rs7161508&chr=14&db=ceu&sz=4&action=1&ht=0&ver=2) | 32752268 | 0.133 | **-3.219** | ND | ND | [rs919521](http://hg-wen.uchicago.edu/cgi-bin/ihh.cgi?target=rs919521&chr=14&db=yri&sz=4&action=1&ht=0&ver=2) | 31277641 | 0.233 | **3.705** | 0.6781 | 1.0647 | [rs8007575](http://hg-wen.uchicago.edu/cgi-bin/ihh.cgi?target=rs8007575&chr=14&db=asn&sz=4&action=1&ht=0&ver=2) | 34612510 | 0.050 | **-3.073** | -0.3605 | 0.1132 |
|  | [rs7161423](http://hg-wen.uchicago.edu/cgi-bin/ihh.cgi?target=rs7161423&chr=14&db=ceu&sz=4&action=1&ht=0&ver=2) | 32752050 | 0.133 | **-3.219** | ND | ND | [rs7149151](http://hg-wen.uchicago.edu/cgi-bin/ihh.cgi?target=rs7149151&chr=14&db=yri&sz=4&action=1&ht=0&ver=2) | 31298293 | 0.175 | **3.701** | 0.6483 | 1.3398 | [rs8012747](http://hg-wen.uchicago.edu/cgi-bin/ihh.cgi?target=rs8012747&chr=14&db=asn&sz=4&action=1&ht=0&ver=2) | 39200076 | 0.400 | **-3.049** | ND | -0.3240 |
|  | [rs1028799](http://hg-wen.uchicago.edu/cgi-bin/ihh.cgi?target=rs1028799&chr=14&db=ceu&sz=4&action=1&ht=0&ver=2) | 32754712 | 0.133 | **-3.206** | ND | ND | [rs4981120](http://hg-wen.uchicago.edu/cgi-bin/ihh.cgi?target=rs4981120&chr=14&db=yri&sz=4&action=1&ht=0&ver=2) | 31270378 | 0.092 | **3.625** | 0.4355 | -0.0812 | [rs1176970](http://hg-wen.uchicago.edu/cgi-bin/ihh.cgi?target=rs1176970&chr=14&db=asn&sz=4&action=1&ht=0&ver=2) | 40505514 | 0.100 | **-3.027** | ND | -0.7572 |
|  | [rs12435308](http://hg-wen.uchicago.edu/cgi-bin/ihh.cgi?target=rs12435308&chr=14&db=ceu&sz=4&action=1&ht=0&ver=2) | 34567833 | 0.200 | **-3.163** | -1.8134 | ND | [rs10129541](http://hg-wen.uchicago.edu/cgi-bin/ihh.cgi?target=rs10129541&chr=14&db=yri&sz=4&action=1&ht=0&ver=2) | 31293590 | 0.158 | **3.596** | 0.5587 | 1.285 | [rs4982053](http://hg-wen.uchicago.edu/cgi-bin/ihh.cgi?target=rs4982053&chr=14&db=asn&sz=4&action=1&ht=0&ver=2) | 32668619 | 0.067 | **-3.009** | ND | ND |
|  | [rs12886242](http://hg-wen.uchicago.edu/cgi-bin/ihh.cgi?target=rs12886242&chr=14&db=ceu&sz=4&action=1&ht=0&ver=2) | 32750225 | 0.133 | **-3.162** | ND | ND | [rs1755778](http://hg-wen.uchicago.edu/cgi-bin/ihh.cgi?target=rs1755778&chr=14&db=yri&sz=4&action=1&ht=0&ver=2) | 35763742 | 0.167 | **3.556** | 1.4941 | 0.9993 | [rs17103033](http://hg-wen.uchicago.edu/cgi-bin/ihh.cgi?target=rs17103033&chr=14&db=asn&sz=4&action=1&ht=0&ver=2) | 34626231 | 0.050 | **-2.995** | 00.1856 | -0.067 |
|  | [rs12882375](http://hg-wen.uchicago.edu/cgi-bin/ihh.cgi?target=rs12882375&chr=14&db=ceu&sz=4&action=1&ht=0&ver=2) | 38886532 | 0.158 | **-3.092** | ND | ND | [rs1169032](http://hg-wen.uchicago.edu/cgi-bin/ihh.cgi?target=rs1169032&chr=14&db=yri&sz=4&action=1&ht=0&ver=2) | 35319325 | 0.183 | **-3.525** | ND | 1.9952 | [rs8011549](http://hg-wen.uchicago.edu/cgi-bin/ihh.cgi?target=rs8011549&chr=14&db=asn&sz=4&action=1&ht=0&ver=2) | 34624884 | 0.050 | **-2.995** | -0.3927 | -0.0670 |
|  | [rs10129933](http://hg-wen.uchicago.edu/cgi-bin/ihh.cgi?target=rs10129933&chr=14&db=ceu&sz=4&action=1&ht=0&ver=2) | 39724743 | 0.183 | **3.083** | 1.0361 | 1.2410 | [rs10148038](http://hg-wen.uchicago.edu/cgi-bin/ihh.cgi?target=rs10148038&chr=14&db=yri&sz=4&action=1&ht=0&ver=2) | 31304269 | 0.725 | **-3.518** | ND | ND | [rs12100841](http://hg-wen.uchicago.edu/cgi-bin/ihh.cgi?target=rs12100841&chr=14&db=asn&sz=4&action=1&ht=0&ver=2) | 34641402 | 0.950 | **2.970** | 0.2700 | -0.1126 |
|  | [rs17102768](http://hg-wen.uchicago.edu/cgi-bin/ihh.cgi?target=rs17102768&chr=14&db=ceu&sz=4&action=1&ht=0&ver=2) | 34354035 | 0.250 | **-3.078** | -0.4351 | -0.5906 | [rs8008432](http://hg-wen.uchicago.edu/cgi-bin/ihh.cgi?target=rs8008432&chr=14&db=yri&sz=4&action=1&ht=0&ver=2) | 31403319 | 0.167 | **3.501** | ND | ND | [rs4899845](http://hg-wen.uchicago.edu/cgi-bin/ihh.cgi?target=rs4899845&chr=14&db=asn&sz=4&action=1&ht=0&ver=2) | 40504963 | 0.089 | **-2.965** | ND | -0.2751 |
|  | [rs17471995](http://hg-wen.uchicago.edu/cgi-bin/ihh.cgi?target=rs17471995&chr=14&db=ceu&sz=4&action=1&ht=0&ver=2) | 32765033 | 0.133 | **-3.077** | ND | ND | [rs8016527](http://hg-wen.uchicago.edu/cgi-bin/ihh.cgi?target=rs8016527&chr=14&db=yri&sz=4&action=1&ht=0&ver=2) | 31392362 | 0.300 | **3.490** | ND | 0.51253 | [rs7151008](http://hg-wen.uchicago.edu/cgi-bin/ihh.cgi?target=rs7151008&chr=14&db=asn&sz=4&action=1&ht=0&ver=2) | 40538564 | 0.089 | **-2.942** | ND | -0.2109 |
|  | [rs12882122](http://hg-wen.uchicago.edu/cgi-bin/ihh.cgi?target=rs12882122&chr=14&db=ceu&sz=4&action=1&ht=0&ver=2) | 32737356 | 0.133 | **-3.076** | ND | ND | [rs1376835](http://hg-wen.uchicago.edu/cgi-bin/ihh.cgi?target=rs1376835&chr=14&db=yri&sz=4&action=1&ht=0&ver=2) | 40519817 | 0.308 | **-3.483** | ND | ND | [rs10137019](http://hg-wen.uchicago.edu/cgi-bin/ihh.cgi?target=rs10137019&chr=14&db=asn&sz=4&action=1&ht=0&ver=2) | 34817787 | 0.950 | **2.907** | -0.1565 | 0.0782 |
|  | [rs1958552](http://hg-wen.uchicago.edu/cgi-bin/ihh.cgi?target=rs1958552&chr=14&db=ceu&sz=4&action=1&ht=0&ver=2) | 33138796 | 0.133 | **3.070** | 0.9891 | 2.4009 | [rs7158021](http://hg-wen.uchicago.edu/cgi-bin/ihh.cgi?target=rs7158021&chr=14&db=yri&sz=4&action=1&ht=0&ver=2) | 31301646 | 0.075 | **3.477** | 0.6483 | 0.3318 | [rs10149823](http://hg-wen.uchicago.edu/cgi-bin/ihh.cgi?target=rs10149823&chr=14&db=asn&sz=4&action=1&ht=0&ver=2) | 34818391 | 0.950 | **2.907** | 0.5080 | ND |
|  | [rs1958549](http://hg-wen.uchicago.edu/cgi-bin/ihh.cgi?target=rs1958549&chr=14&db=ceu&sz=4&action=1&ht=0&ver=2) | 33134526 | 0.233 | **3.040** | 1.1647 | 2.8621 | [rs12433539](http://hg-wen.uchicago.edu/cgi-bin/ihh.cgi?target=rs12433539&chr=14&db=yri&sz=4&action=1&ht=0&ver=2) | 31401393 | 0.158 | **3.470** | ND | ND | [rs7218](http://hg-wen.uchicago.edu/cgi-bin/ihh.cgi?target=rs7218&chr=14&db=asn&sz=4&action=1&ht=0&ver=2) | 34812814 | 0.950 | **2.905** | -0.4428 | -0.4388 |
| ***Total negative values*** |  |  |  | **14** |  |  |  |  |  | **6** |  |  |  |  |  | **13** |  |  |
| ***%*** |  |  |  | **70** |  |  |  |  |  | **30** |  |  |  |  |  | **65** |  |  |
| ^a^iHS values recovered from the Haplotter( http://plab-server.uchicago.edu/selection/) and SNP@evolution (http://bighapmap.big.ac.cn/) databases. | | | | | | | | | | | | | | | | | | |
