# Supplementary material for: Genetic Variation among Major Human Geographic Groups Supports a Peculiar Evolutionary Trend in PAX9
Source: PLoS One. 2011 Jan 27;6(1):e15656. doi: 10.1371/journal.pone.0015656 (PMC3029280; doi:10.1371/journal.pone.0015656)
Supplement: Table S6 — Sample sizes, geographic location, and linguistic information on fifteen South Amerindian, one Eskimo and one composite African populations investigated in this study (DOC) [file pone.0015656.s006.doc]

| **Table S6.** Sample sizes, geographic location, and linguistic information on fifteen South Amerindian, one Eskimo and one composite African populations investigated in this study | | | | | |
| --- | --- | --- | --- | --- | --- |
| Populations | No. of individuals | Geographic location | Country/Region | Language classification^a^ | References for additional information |
| South Amerindians |  |  |  |  |  |
| Aché | 20 | 23^o^30´- 56 ^o^30´W | Paraguay | Tupian | [3-6] |
| Apalaí | 9 | 1 ^o^ 20´N - 54 ^o^ 40´W | Brazil/North | Cariban | [7, 8] |
| Arara | 21 | 3 ^o^ 30´ S - 54 ^o^ 10´W | Brazil/North | Arara | [9] |
| Bari | 4 | 12 ^o^ 50´S - 73 ^o^ 5´W | Venezuela | Chibchan | [6] |
| Cayapo | 1 | 10 ^o^ 20´S - 53 ^o^ 5´W | Brazil/North | Jêan | [10] |
| Guarani | 5 | 23 ^o^ 6´ S-55 ^o^ 12 W | Brazil/South | Tupian | [6, 11, 12] |
| Galibi | 7 | 3 ^o^ 45´N - 51 ^o^ 15´W | Brazil/North | Cariban | [13] |
| Jamamadi | 5 | 7 ^o^ 15´S - 66 ^o^ 41´W | Brazil/North | Arahuan | [9] |
| Kaingang | 20 | 27 ^o^ 20´S - 52 ^o^ 45´W | Brazil/South | Jêan | [6, 12, 14, 15] |
| Mekranoti | 5 | 8 ^o^ 40´S - 54 ^o^ W | Brazil/North | Jêan | [10] |
| Mundurucu | 8 | 6 ^o^ 23´S - 59 ^o^ 9´W | Brazil/North | Tupian | [9] |
| Tenharim | 8 | 8 ^o^ 20´S - 54 ^o^ W | Brazil/North | Tupian | [13] |
| Warao | 4 | 11 ^o^ 50´S - 74 ^o^ 5´W | Venezuela | Warao | [6] |
| Xikrin | 17 | 5 ^o^ 55´S - 51 ^o^ W | Brazil/North | Jêan | [10] |
| Yucpa | 4 | 12 ^o^ 50´S - 71^o^ 5´W | Venezuela | Chibchan | [6] |
|  |  |  |  |  |  |
| Northern Arctic |  |  |  |  |  |
| Eskimo | 44 | 64 ^o^ N - 175 ^o^ W | Russia | Eskimo-Aleut | [16-18] |
|  |  |  |  |  |  |
| Africa |  |  |  |  |  |
| African^b^ | 43 |  | Democratic Republic of Congo, Cameroon, Ivory Coast | Niger-Congo | [19, 20] |
| ^a^Acording to Greenberg [1] and Campbell [2].  ^b^ A composite sample from Bantu and non-Bantu speakers living in the Democratic Republic of Congo, Cameroon, and Ivory Coast, while four additional individuals were ascertained in Paris, France. | | | | | |

**References Supplementary Material**

1. Greenberg JH (1963) The language of Africa. Bloomington: Indiana University.

2. Campbell L (1997) American Indian languages: the historical linguistics of Native America, New York: Oxford University Press.

3. Brown SM, Gajdusek DC, Leyshon WC, Steinberg AG, Brown KS, et al. (1974) Genetic studies in Paraguay: blood group, red cell, and serum genetic patterns of the Guayaki and Ayore Indians, Mennonite settlers, and seven other Indian tribes of the Paraguayan Chaco. Am J Phys Anthropol 41: 317–343.

5. Hill K, Hurtado M (1996) Ache life history: the ecology and demography of a foraging people. New York: Aldine de Gruyter.

6. Bortolini MC, Salzano FM, Thomas MG, Stuart S, Nasanen SP, et al. (2003) Y-chromosome evidence for differing ancient demographic histories in the Americas. Am J Hum Genet 73: 524-539.

7. Salzano FM, Black FL, Callegari-Jacques SM, Santos SE, Weimer TA, et al. (1988) Genetic variation within a linguistic group: Apalai-Wayana and other Carib tribes. Am J Phys Anthropol 75: 347-356.

8. Mazières S, Guitard E, Crubézy E, Dugoujon JM, Bortolini MC, et al (2008) Uniparental. mtDNA, Y-chromosome. polymorphisms in French Guiana and two related populations--implications for the region's colonization. Ann Hum Genet 72: 145-156.

9. Salzano FM, Black FL, Callegari-Jacques SM, Santos SE, Weimer TA, et al. (1991) Blood genetic systems in four Amazonian tribes. Am J Phys Anthropol. 85: 51-60.

10. Salzano FM, Neel JV, Weitkamp LR, Woodall JP (1972) Serum proteins, hemoglobins and erythrocyte enzymes of Brazilian Cayapo Indians. Hum Biol 44: 443-458.

11. Salzano, FM, Callegari-Jacques SM, Weimer TA, Franco MHLP, Hutz MH, et al (1997) Electrophoretic protein polymorphisms in Kaingang and Guarani Indians of southern Brazil. Am J Hum Biol 9: 505–512.

12. Marrero AR, Silva-Junior WA, Bravi CM, Hutz MH, Petzl-Erler ML, et al. (2007) Demographic and evolutionary trajectories of the Guarani and Kaingang natives of Brazil. Am J Phys Anthropol 132: 301-310.

13. Santos SEB, Ribeiro-Dos-Santos AK, Guerreiro JF, Santos EJ, Weimer TA, et al. (1998) New protein genetic studies in six Amazonian Indian populations. Ann Hum Biol 25: 505-522.

14. Salzano FM, Callegari Jacques SM, Franco MH, Hutz MH, Weimer TA, et al. 1980. The Caingang revisited: blood genetics and anthropometry. Am J Phys Anthropol 53: 513-524.

15. Belich MP, Madrigal JA, Hildebrand WH, Zemmour J, Williams RC et al. (1992) Unusual HLA-B alleles in two tribes of Brazilian Indians. Nature 357: 326-329.

16. Erdesz S, Shubin SV, Shoch BP, Krylov My, Mylov NM et al. (1994) Spondyloarthropathies in circumpolar populations of Chukotka. Eskimos and Chukchi): epidemiology and clinical characteristics. J Rheumatol 21: 1101-1104.

17. Krylov M, Erdesz S, Alexeeva L, Benevolenskaya L, Arnett FC, et al. (1995) HLA class II and HLA-B27 oligotyping in two Siberian native population groups. Tissue Antigens 46: 382-386.

18. Krylov MIu, Erdes SH, Alekseeva LI, Benevolenskaia LI (1995) DNA typing of HLA class II genes among the aboriginal inhabitants of Chukotka. Genetika 31: 852-858.

19. Silva WA Jr, Bortolini MC, Meyer D, Salzano FM, Elion J, et al. (1999) Genetic diversity of two African and sixteen South American populations determined on the basis of six hypervariable loci. Am J Phys Anthropol 109: 425-437.

20. Silva WAJr, Bortolini MC MC, Schneider MPC, Marrero AR, Elion J, et al. (2006) mtDNA haplogroups analysis of Black Brazilain ans sub-Saharan populations: implications for the Atlantic slave trade. Hum Biol 78: 29-41.
